# Keep it simple: Intimal calcification for predicting atherosclerotic large vessel occlusion

**DOI:** 10.1111/ene.70028

**Published:** 2024-12-30

**Authors:** Marialuisa Zedde, Rosario Pascarella

**Affiliations:** ^1^ Neurology Unit, Stroke Unit Azienda Unità Sanitaria Locale‐IRCCS di Reggio Emilia Reggio Emilia Italy; ^2^ Neuroradiology Unit Azienda Unità Sanitaria Locale‐IRCCS di Reggio Emilia Reggio Emilia Italy

Intracranial atherosclerotic disease (ICAD) is a major cause of ischemic stroke worldwide, being more common than previously thought in Caucasian patients too [1]. Symptomatic ICAD is an aggressive entity with a very high risk of recurrent ischemic events, despite best medical therapy [2]. Substantial uncertainty remains as which are the best treatments for this disease, especially for high‐risk patients. The rapidly increasing use of cerebral vascular imaging to guide therapeutic decisions in the hyperacute phase of stroke is making ICAD recognition highly accessible for a growing number of patients, both as a potential cause of the ischemic event and coexisting disease [2].

ICAD can be the underlying cause of large vessel occlusion (LVO) in stroke patients undergoing mechanical thrombectomy (MT). The diagnosis of ICAD in this population can be very challenging and it can be suspected with high probability attending to the characteristics and behavior of the artery at the site of occlusion during MT. Some of these imaging features have been retrospectively validated using computed tomography (CT) angiography too, but their reliability is a matter of discussion, in particular in non‐Asian populations [2, 3]. Three surrogate markers for ICAD‐related occlusion have been reported: fixed focal stenosis (FFS), truncal‐type occlusion (TTO), and branching‐site occlusions (BSO) [2, 3]. Usually, a TTO is seen on the initial Digital Subtraction Angiography (DSA) series. Then, suboptimal arterial opening with residual stenosis is frequently observed while opening the stent or after several stent‐retriever passes. Another typical feature is the early worsening of arterial caliber after thrombectomy, which can lead to arterial reocclusion [4]. In the available studies [2, 3, 4], the likelihood that this refractoriness to thrombectomy is caused by ICAD increases when other causes of LVO are lacking. These last issues are, for example, the absence of a known major cardiac embolic source, preceding transitory symptoms that can be explained by ischemia in the same arterial territory, absence of CT arterial hyperdense sign or magnetic resonance imaging (MRI) susceptibility sign, or watershed‐type infarction suggesting hemodynamic compromise caused by a preexisting stenotic lesion. In most cases, the diagnosis of ICAD‐related LVO is probabilistically made during MT and not before it. Unfortunately, this issue may affect the choice of the thrombectomy strategy.

The ideal scenario is to define that LVO is ICAD‐related before thrombectomy and several ways, that is, measuring the Hounsfield Units of a nonhyperdense clot, have been proposed with unsatisfying results. One of the last proposals is about perfusion parameters, finding that an increased or normal cerebral blood volume (CBV) pattern may be associated with ICAD LVO on the pretreatment perfusion imaging [5]. This proposal is interesting and it refers to a preconditioning of the territory supplied by the occluded artery, assuming a precedent hemodynamic stenosis in the same artery. Unfortunately, the data supporting this proposal were retrospective and based on MRI, which is not the most widely available technique worldwide in acute stroke management.

Sierra‐Gómez et al. [6] addressed pragmatically this issue in acute stroke treatment, trying to early define the ICAD‐related LVO in order to have prognostic information and to guide the endovascular procedure. They used a simple and efficient diagnostic technique, that is, noncontrast CT (NCCT), read using the bone window, without adding more investigations in the diagnostic flow of a time‐dependent disease. The premise of this proposal is simple and easy. Intracranial arterial calcifications are a common incidental finding on CT imaging in the general population, but the distribution of calcifications in different large and small arterial beds as well as the location in the intimal or medial layer of the arterial wall might imply different risk factors and clinical outcomes. Concentric calcifications occur in the tunica media and result in arterial stiffening, but they are not related to atherosclerosis, which is an intimal disease. Moreover, intimal and medial calcification of intracranial arteries can be distinguished in CT and this technique is validated against hystopathology [7]. The authors6 fundamentally applied the intracranial internal carotid artery calcification (iICA) score to an NCCT image obtained using a 64‐slice multidetector row CT system with 1.25 mm slice thickness. The first advantage is that the technique is reproducible even in relatively low‐resource settings. In addition, they demonstrated that the atherosclerotic type intracranial calcifications are associated with a worse functional outcome and a larger infarct volume6. This worse prognosis might be related to different factors (refractoriness to thrombectomy due to vessel tortuosity, thrombus composition, etc.), but it is not yet clear which mechanism is prevalent in the described cohorts.

As pointed out by the authors [6], the iICA calcium score is accessible, easy to apply in the acute phase of stroke, and validated versus histopathology, making it proposable for a larger validation. It is a subjective tool, then, it is not clear if training is needed before applying it, but, if so, it could be as easy as ASPECTS for vascular neurologists and neuroradiologists involved in stroke treatment.

## AUTHOR CONTRIBUTIONS

**Marialuisa Zedde:** Conceptualization; methodology; writing – original draft; writing – review and editing. **Rosario Pascarella:** Conceptualization; methodology; writing – original draft; writing – review and editing.

## FUNDING INFORMATION

No funding was received to assist with the preparation of this manuscript.

## CONFLICT OF INTEREST STATEMENT

The authors declare no conflicts of interest.

## Data Availability

Data sharing not applicable to this article as no datasets were generated or analysed during the current study.
